# miR-221-3p Regulates VEGFR2 Expression in High-Risk Prostate Cancer and Represents an Escape Mechanism from Sunitinib In Vitro

**DOI:** 10.3390/jcm9030670

**Published:** 2020-03-02

**Authors:** Markus Krebs, Antonio Giovanni Solimando, Charis Kalogirou, André Marquardt, Torsten Frank, Ioannis Sokolakis, Georgios Hatzichristodoulou, Susanne Kneitz, Ralf Bargou, Hubert Kübler, Bastian Schilling, Martin Spahn, Burkhard Kneitz

**Affiliations:** 1 Department of Urology and Pediatric Urology, University Hospital Würzburg, 97080 Würzburg, Germany; kalogirou_c@ukw.de (C.K.); torsten.frank@stud-mail.uni-wuerzburg.de (T.F.); kuebler_h@ukw.de (H.K.); 2 Comprehensive Cancer Center Mainfranken, University Hospital Würzburg, 97080 Würzburg, Germany; bargou_r@ukw.de; 3 IRCCS Istituto Tumori “Giovanni Paolo II” of Bari, Viale Orazio Flacco, 65, 70124 Bari, Italy; antoniogiovannisolimando@gmail.com; 4 Department of Biomedical Sciences and Human Oncology, Section of Internal Medicine “G. Baccelli”, University of Bari Medical School, 70124 Bari, Italy; 5 Institute of Pathology, University of Würzburg, 97080 Würzburg, Germany; marquardt_a@ukw.de; 6 Interdisciplinary Center for Clinical Research, University Hospital Würzburg, 97080 Würzburg, Germany; 7 Department of Urology, Martha-Maria Hospital Nuremberg, 90491 Nuremberg, Germany; ioannis.sokolakis@martha-maria.de (I.S.); georgios.hatzichristodoulou@martha-maria.de (G.H.); 8 Chair of Physiological Chemistry, University of Würzburg, 97074 Würzburg, Germany; susanne.kneitz@uni-wuerzburg.de; 9 Department of Dermatology, University Hospital Würzburg, 97080 Würzburg, Germany; Schilling_B@ukw.de; 10 Department of Urology, Lindenhofspital Bern, 3012 Bern, Switzerland; martin.spahn@lindenhofgruppe.ch

**Keywords:** microRNA-221, high-risk Prostate Cancer, angiogenesis, Sunitinib, Tyrosine kinase inhibition

## Abstract

Downregulation of miR-221-3p expression in prostate cancer (PCa) predicted overall and cancer-specific survival of high-risk PCa patients. Apart from PCa, miR-221-3p expression levels predicted a response to tyrosine kinase inhibitors (TKI) in clear cell renal cell carcinoma (ccRCC) patients. Since this role of miR-221-3p was explained with a specific targeting of VEGFR2, we examined whether miR-221-3p regulated VEGFR2 in PCa. First, we confirmed VEGFR2/KDR as a target gene of miR-221-3p in PCa cells by applying Luciferase reporter assays and Western blotting experiments. Although VEGFR2 was mainly downregulated in the PCa cohort of the TCGA (The Cancer Genome Atlas) database, VEGFR2 was upregulated in our high-risk PCa cohort (*n* = 142) and predicted clinical progression. In vitro miR-221-3p acted as an escape mechanism from TKI in PC3 cells, as displayed by proliferation and apoptosis assays. Moreover, we confirmed that Sunitinib induced an interferon-related gene signature in PC3 cells by analyzing external microarray data and by demonstrating a significant upregulation of miR-221-3p/miR-222-3p after Sunitinib exposure. Our findings bear a clinical perspective for high-risk PCa patients with low miR-221-3p levels since this could predict a favorable TKI response. Apart from this therapeutic niche, we identified a partially oncogenic function of miR-221-3p as an escape mechanism from VEGFR2 inhibition.

## 1. Introduction

Neo-angiogenesis constitutes a crucial event for cancer progression. For prostate cancer (PCa), abundant angiogenic signalling has been associated with aggressive courses of disease [1,2]. Specifically, VEGFR2, which is one of the main therapeutic targets of tyrosine kinase inhibitors (TKI), was reported to be upregulated in aggressive PCa [3,4]. While TKI-based regimens do not appear promising for unselected PCa patients at first sight [5], distinct patient subgroups could benefit from such a treatment. 

For clear-cell renal cell carcinoma (ccRCC), the tissue expression of miR-221-3p and miR-222-3p, which are two small, non-coding RNA strands originating from the same cluster, significantly predicted the response towards antiangiogenic therapies in two independent studies [6,7]. Functionally, Khella et al. reasoned that this trait was associated with miR-221-3p/miR-222-3p specifically binding the mRNA of KDR/VEGFR2, which decreases VEGFR2 protein levels [7]. 

In case of PCa, miR-221-3p and miR-222-3p are significantly downregulated in malignant tissue when compared to benign tissue, as confirmed by previous studies [8,9,10] including a recent meta-analysis [11]. Our study group was among the first demonstrating this downregulation in a high-risk PCa cohort [8]. Progressive downregulation of miR-221-3p significantly predicted cancer-specific as well as overall survival in two independent PCa cohorts [12]. On a molecular basis, there are conflicting data regarding miR-221-3p function in PCa cells. While our group could show that restoration of miR-221-3p expression had a tumour suppressive role in castration-resistant DU145 and PC3 cells [12], other researchers demonstrated that miR-221-3p also acted as an oncogene in LNCaP and PC3 cells by supporting progress to a castration-resistant state [13,14,15,16].

Regarding the prognostic role of miR-221-3p/miR-222-3p expression in the TKI response of ccRCC patients and the downregulation of miR-221-3p/miR-222-3p in PCa tissue, we wanted to find out whether miR-221-3p also regulated VEGFR2 in PCa cells. Lastly, we aimed to assess the influence of miR-221-3p expression on the TKI sensitivity of PCa cells. 

## 2. Materials and Methods

### 2.1. Cell Culture

PC3 and LNCaP cells were obtained from ATCC (American Type Culture Collection, Chicago, IL, USA) and cultured in RPMI 1640 from PAALaboratories (Pasching, Austria) supplemented with 10% fetal calf serum (FCS) and 2 mMol Glutamine. Cells were maintained in a 5% CO_2_ incubator at 37 °C.

### 2.2. RNA Extraction, Reverse Transcription, and qRT-PCR

Total RNA was extracted from cell lines 48 h after transient transfection (p. t.) of miR-221 using TRIzol reagent (Life Technologies, Carlsbad, CA, USA). RNA from paraffin-embedded PCa tissues was extracted as described previously [12]. The RNA concentration was determined with a bioanalyzer (Agilent, Santa Clara, CA, USA). cDNA was synthesized from total RNA with stem-loop reverse transcription primers, according to the TaqMan miR-assay protocol (Life Technologies, Carlsbad, CA, USA) or the Promega ImProm II reverse transcription system (Promega, Madison, WI, USA). Mature miR-expression was quantified with the TaqMan miR-221 assay kit and an Applied Biosystems 7900 HT system. We followed the protocol provided in the manufacturer’s instructions (Life Technologies, Carlsbad, CA, USA). The expression of small nuclear RNA (snRNA) RNU6b was used for normalization. Relative miR expression was calculated with the comparative ∆Ct-method (∆Ct sample = Ct sample − Ct RNU6b). Fold changes in miR expression between samples and controls were determined by the 2^∆∆Ct^ method (in this study, referred to as the ∆∆Ct method). mRNA expression analysis of VEGFR2/KDR expression was performed according to standard qRT-PCR procedures. Primer sequences are available upon request. Mean Ct was always determined from triplicate PCRs.

### 2.3. Luciferase Reporter Assays and DNA Construction

Plasmids used and the procedure of cloning were described previously [17]. To amplify the putative miR-221 binding site at the 3’UTR of human VEGFR2/KDR gene, we designed specific primers. HindIII sequences were added to the end of the specific primer pairs to clone the resulting VEGFR2 fragment into the HindIII site of the Luciferase reporter plasmid (pMIR-REPORT-Luciferase, Applied Biosystems). In addition, we introduced a mutation into the miR-221 binding site of the VEGFR2/KDR fragment using the Site-Directed Mutagenesis Kit (QuickChange, Agilent Technologies) and introduced the mutant fragment into the Luciferase reporter plasmid. This mutant reporter vector was used to confirm binding of miR-221 to its putative binding site at the 3’UTR of human VEGFR2/KDR. All primer sequences are available upon request. Transient transfection and Luciferase assays were performed as described previously [17]. A non-targeting miRNA oligonucleotide was used as a control. All reporter assays were repeated at least four times. 

### 2.4. Western Blotting Experiments

After harvesting with ethylenediaminetetraacetic acid (EDTA), cells were washed twice with phosphate-buffered saline (PBS) and lysed in Novagen’s PhosphoSafe (Merck, Darmstadt, Germany) following manufacturer’s instructions. Total protein concentrations were quantified (Bradford reagent). Protein isolates were loaded on 12.5% SDS-PAGE gels with a concentration of 50 ng per lane and transferred to nitrocellulose membranes (Bio-Rad, Hercules, CA, USA). The membranes were blocked using block-buffer (Invitrogen, Carlsbad, CA, USA) and incubated at 4 °C with the primary antibody following the manufacturers’ instructions. For protein expression by Western blot analysis, we used the following antibodies: VEGFR2 (Abcam) and ERK-2 (Ambion) as a loading control. We used horseradish peroxidase–coupled secondary antibodies and the Amersham ECL Prime reagent (GE Healthcare, Little Chalfont, United Kingdom) to visualize the protein expression.

### 2.5. Patient Samples

PCa tissue from radical prostatectomy (RP) specimen (*n* = 142) was obtained from the Department of Urology at the Community Hospital Karlsruhe, Germany. Samples were paraffin-embedded. Fractions with >90% cancerous tissue were used. All patients were recruited from a well-characterized group of high-risk PCa patients of the EMPaCT tumor bank (European Multicenter Prostate Cancer Clinical and Translational Research Group) as described previously [8,12]. According to the high-risk PCa criteria established by D’Amico et al. [18], all patients had a preoperative/initial serum prostate-specific antigen (PSA) of at least 20 µg/L. Clinical progression was declared when either local or distant metastases were histologically proven or confirmed by CT or the bone scan. The study was approved by the local ethics committee (no. 59/04 in 2004) and all patients provided written informed consent. miR-221 expression analysis data of *n* = 118 patient samples were used from a previous study [12]. Table 1 shows the basic characteristics of our study cohort.

### 2.6. Proliferation Assays (MTS) and Transfection

PC3 cells were cultured at 2 ×10^3^ cells per well and LNCaP cells were cultured at 1 × 10^4^ cells per well in triplicates in 96-well plates (Greiner Bio-One, Frickenhausen, Germany). Transient transfections with human precursor miR-221 (pre-miR-221) and the respective controls (negative control oligonucleotide, pre-miR-Ctr) were carried out using Lipofectamine following the manufacturer´s instructions (Applied Biosystems, Waltham, MA, USA), as described previously [12]. Sunitinib Malate and the specific VEGFR2 inhibitor Ki8751 were both obtained from SelleckChem. 10 µmol of Sunitinib Malate as well as 10 µmol Ki8751 was administered for 24 h p. t. Lastly, the MTS assays were performed 72 h p. t. Cells were analyzed with MTS CellTiter96 Proliferation Assay (Promega, Madison, WI, USA) at 490 nm with a monochromator (Bio-Rad, Hercules, CA, USA) following the manufacturer’s protocol.

### 2.7. Apoptosis Assays

For assessing apoptosis in vitro, we analyzed Caspase 3/7 activity by applying the Caspase-GLO 3/7 Kit (Promega, Madison, WI, USA), as described earlier [12]. As delineated in the paragraphs above, cells were transfected with pre-miR-221 or corresponding ctr-RNAs and Sunitinib (10 µM) was administered 36 h p. t. After incubation with Caspase 3/7 reagent for 4 h at room temperature, lysed cells were transferred to white-walled 96-well plates for measuring luminescence. These steps were carried out according to the manufacturer’s protocol. We carried out three independent experiments. Each single experiment was performed by analyzing triplicate measurements.

### 2.8. Statistical Analysis/Software

We used R build 3.2.2 (R foundation, Vienna, Austria) for statistical evaluation. If not stated otherwise, the Student’s unpaired t-test was used to discriminate significant differences in normally distributed data. Significance levels were determined as α = 95% and α = 99%. Statistically significant associations were set as * *p* < 0.05, ** *p* < 0.01, and *** *p* < 0.001. For microRNA target prediction, we used the targetscan.org web resource [19]. External microarray data from Sunitinib-treated PC3 cells were downloaded via L1000 fireworks database [20]. For further analyses of overexpressed genes within external microarray data, we used the Enrichr web application [21,22] in order to apply *Reactome 2016* analysis [23]. 

Several results presented in this manuscript are partly based upon data generated by the TCGA (The Cancer Genome Atlas) Research Network (see Acknowledgements). Table 2 outlines the characteristics of the PCa study cohort within the TCGA database. Other solid tumor entities with significantly aberrant VEGFR2 expression within the TCGA database were collected using the UALCAN web tool [24]. VEGFR2 expression data within the Dream Team cohort [25] of PCa metastases were accessed by using cbioportal.org [26]. 

## 3. Results

### 3.1. VEGFR2 as a Direct Target Gene of miR-221-3p in PCa Cells

**Figure 1:**
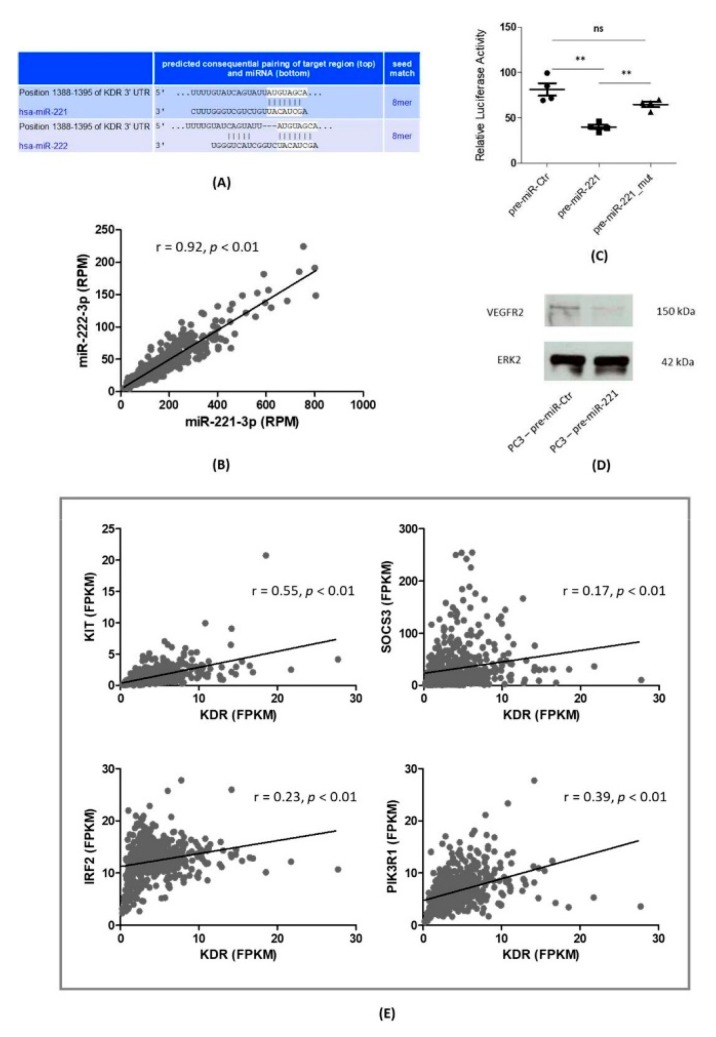
VEGFR2/KDR is a direct target gene of miR-221-3p in PCa. (**A**) Bioinformatically predicted KDR targeting sites (TargetScan.com) for miR-221-3p and miR-222-3p. (**B**) Markedly positive correlation for miR-221-3p and miR-222-3p expression within the PCa cohort of the TCGA (The Cancer Genome Atlas) database. RPM: reads per million. (**C**) Luciferase reporter assays confirmed a direct binding of miR-221-3p and VEGFR2: transfection with pre-miR-221-3p led to a highly significant decrease in relative Luciferase activity. Mutation of the predicted binding site significantly restored relative Luciferase activity. Results shown here consist of four independent experiments. ns: not significant. **: *p* < 0.01. (**D**) Elevated cellular miR-221-3p levels led to a diminished protein expression of VEGFR2 in PC3 cells 48 h after transient transfection (p. t.) kDa: kilodalton. (**E**) Positive correlations (r = Pearson rank correlation coefficients) of KDR and the established miR-221-3p target genes KIT, IRF2, SOCS3, and PIK3R1. FPKM: fragments per kilobase million.

At first, we were looking for bioinformatically predicted target sites of miR-221-3p/miR-222-3p and VEGFR2/KDR. Using the Targetscan.com web resource [19] revealed closely-related binding sites for both miRs, which originate from the same gene cluster (Figure 1A). Moreover, analyzing the expression levels of miR-221-3p and miR-222-3p within the PCa cohort of the TCGA database (Figure 1B) revealed a Pearson correlation coefficient of r = 0.92 (*p* < 0.01). Based on this result, we concluded that the expression levels of miR-221-3p and miR-222-3p are tightly linked in PCa tissue. In the following part of the manuscript, we used the short descriptions miR-221/miR-222 instead of miR-221-3p/miR-222-3p. Next, we performed Luciferase reporter assays using the bioinformatically predicted targeting site within the three prime untranslated regions (3’UTR) of the VEGFR2/KDR mRNA. As shown in Figure 1C, pre-miR-221 co-transfections resulted in a highly significant reduction of relative Luciferase activity (39.97 ± 4.88 RLU, *p* = 0.0059) compared to control co-transfections (81.35 ± 13.8 RLU). Furthermore, co-transfection of miR-221 with a plasmid carrying a mutated VEGFR2/KDR binding site nearly restored the initial relative Luciferase activity and did not show significant changes in Luciferase activity compared to control co-transfections (64.83 ± 12.74 RLU, *p* = 0.09). Moreover, Western blotting experiments confirmed a downregulation of the VEGFR2 protein in pre-miR-221-transfected PC3 cells when compared to control transfections (Figure 1D). Further gene expression analyses within the TCGA database (Figure 1E) revealed significantly positive Pearson correlation coefficients of KDR and previously established miR-221 target genes in PCa such as KIT, IRF2, SOCS3, and PIK3R1.

### 3.2. VEGFR2 Expression in PCa Tissue

Regarding VEGFR2 expression in the PCa specimen, we primarily examined the PCa cohort within the TCGA database. Remarkably, VEGFR2 was downregulated in malignant tissue in comparison to surrounding benign tissue. As illustrated in Table 3, PCa was not the only solid tumor entity with VEGFR2 reported to be downregulated. Instead, a majority of malignancies—among them, Lung Adenocarcinoma, papillary RCC, and Urothelial Carcinoma—showed significantly lower VEGFR2 levels in cancer specimen. Glioblastoma, ccRCC, and Stomach adenocarcinoma were the only entities reported to significantly overexpress VEGFR2 when compared to benign tissue. Regarding patients´ survival, only VEGFR2 upregulation in ccRCC (favorable prognosis) and downregulation in papillary RCC (favorable prognosis) had a significant impact in the TCGA database.

**Figure 2:**
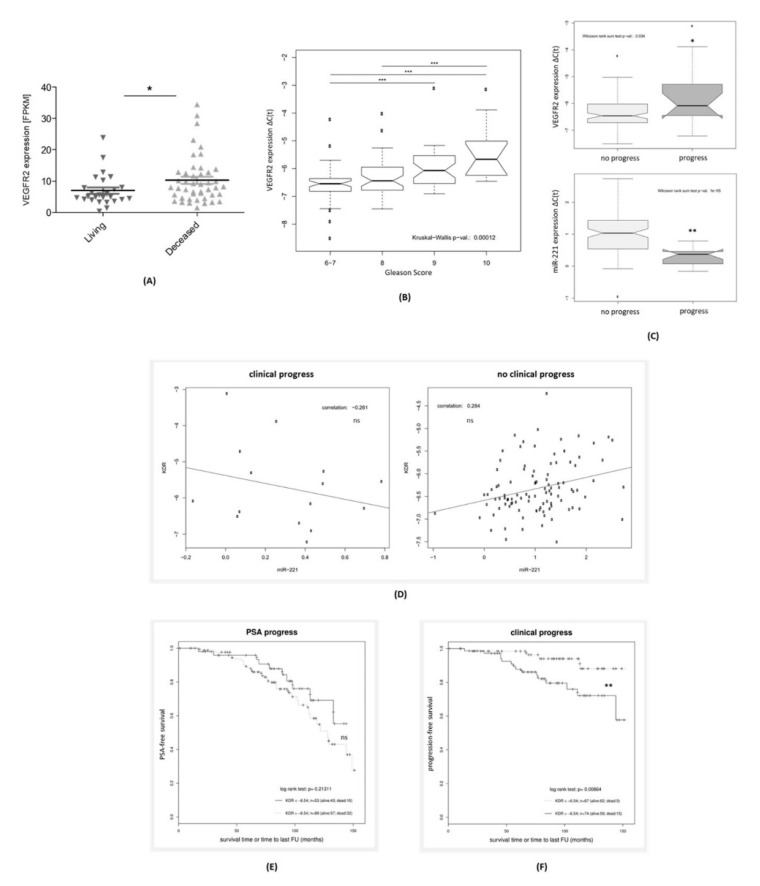
Relative upregulation of VEGFR2 in the Dream Team database of metastatic PCa and our in-house high-risk PCa cohort. (**A**) Significant upregulation of VEGFR2 expression within PCa metastases from patients deceased within the follow-up period (Dream Team database, accessed via cbioportal.org). (**B**) Significant rise of VEGFR2 expression in radical prostatectomy (RP) specimen with higher Gleason Scores in an in-house high-risk PCa cohort (*n* = 142). (**C**) VEGFR2 and miR-221 expression in PCa tissue according to the endpoint of clinical progress. (**D**) Pearson correlation coefficients for miR-221 and VEGFR2 expression in high-risk PCa specimen depending on progression. (**E**,**F**) Kaplan Meier plots of VEGFR2 expression for PSA progress (**E**) and Clinical progress (**F**). (A–F) ns: not significant. *: *p* < 0.05. **: *p* < 0.01. ***: *p* < 0.001.

Yet, in contrast to the VEGFR2 downregulation in the TCGA database by containing a high proportion of low and intermediate risk PCa, searching the Dream Team cohort of PCa metastases [25] for VEGFR2 expression revealed a significant upregulation in the deceased compared to surviving patients (*p* < 0.05, Figure 2A). This led us to analyze the stage-dependent VEGFR2 expression levels within our in-house high-risk PCa cohort consisting of *n* = 142 RP specimen. Figure 2B–F illustrates our results. VEGFR2 expression rose with higher Gleason scores in a highly significant manner (Figure 2B) among high-risk PCa samples. We additionally analyzed VEGFR2 and miR-221 levels depending on the endpoint. Regarding clinical progress, while PCa patients suffering from progression had significantly higher VEGFR2 levels (*p* = 0.034), miR-221 levels in these patients decreased in a highly significant manner (*p* = 0.00001, Figure 2C), as described previously [8,12]. 

Regarding Pearson correlation coefficients of miR-221 and VEGFR2 expression in our database (Figure 2D), we discovered diverging results depending on the criterion of clinical progress. While PCa tissue from patients without clinical progression showed a positive correlation coefficient of r = 0.284 (not significant), miR-221 and VEGFR2 were negatively correlated in the patient subgroup suffering from a clinical progression within the follow-up period (r = -0.261, not significant). For PSA progress, the following Kaplan Meier analyses (Figure 2E) revealed a shorter PSA-free survival for PCa patients with high VEGFR2 levels. Yet, this fact did not result in a significant prognostic potential (*p* = 0.213). However, higher VEGFR2 levels significantly predicted a shorter progression-free survival (*p* = 0.0086, Figure 2F) in our high-risk PCa cohort.

### 3.3. miR-221-3p Upregulation as an Escape Mechanism from VEGFR2 Inhibition in PC3 Cells

**Figure 3:**
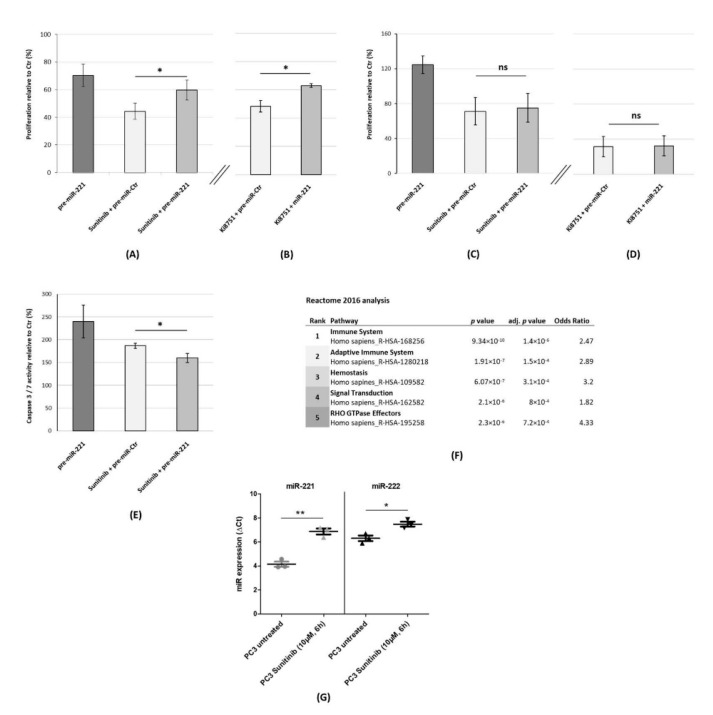
Re-expression of miR-221 as part of an escape mechanism from VEGFR2 inhibition in PC3 cells. (**A**) Elevation of cellular miR-221 levels (by pre-miR-221 transfection) counteracts the effect of Sunitinib administration. (**B**) Restoration of cellular miR-221 expression is associated with a higher proportion of viable cells after Ki8751 treatment. (**A**,**B**) PC3 cells were transiently transfected with pre-miR-221 or pre-miR-Ctr. Sunitinib (10 µM) or Ki8751 (10 µM) were administered 24 h p. t. MTS assays were performed 72 h p. t. (**C**,**D**). Pre-miR-221 transfection did not significantly alter the response towards Sunitinib (**C**) and Ki8751 (**D**) in castration-sensitive LNCaP cells. TKI (both 10 µM) were administered 24h p. t. The MTS assays were performed 72 h p. t. (**E**) miR-221 overexpression significantly lowered apoptosis induction after Sunitinib treatment (10 µM; 12 h), as indicated by relative Caspase 3/7 activity. (**F**) Reactome 2016 analysis of publicly available microarray data revealed an interferon-related / immune-mediated signature in PC3 cells after Sunitinib exposure (10 μM, 6 h). (**G**) Significant upregulation of miR-221 and miR-222 expression after Sunitinib treatment (10 μM, 6 h) in PC3 cells. (**A–G**) ns: not significant. *: *p* < 0.05. **: *p* < 0.01.

Since VEGFR2 is one of the main target structures of TKI therapies, we also looked at the interaction of miR-221 expression and Sunitinib response. As illustrated in Figure 3A, administration of Sunitinib (10 µM) caused an inhibition of proliferation in PC3 cells (44.33 ± 5.86% viable cells 72 h p. t. relative to control transfections), whereas overexpression of miR-221 in combination with Sunitinib diminished the antiproliferative effect of Sunitinib significantly (59.66 ± 7.23% viable PC3 cells 72 h p. t. relative to control transfections, *p* = 0.048). This result indicates that high miR-221 levels reduced the sensitivity of PC3 cells toward the antiproliferative effect of Sunitinib.

Given that this de-sensitizing effect could also be mediated by other Sunitinib target genes like KIT and Platelet-derived growth factor receptor (PDGFR), we also employed Ki8751 (10 µM), which is a specific inhibitor of VEGFR2 (Figure 3B). We observed a significant miR-221-mediated desensitization of PC3 cells toward specific VEGFR2 inhibition (*p* = 0.018) from 48.3 ± 4.14% viable cells in Ki8751-treated, control-transfected cells to 62.95 ± 1.29% after a combination of pre-miR-221 transfection and Ki8751 treatment. In conclusion, miR-221 overexpression as part of an escape mechanism from TKI is not exclusive to Sunitinib treatment. 

We also analyzed the influence of miR-221 on the TKI response in LNCaP cells. While pre-miR-221 transfection—in contrast to PC3 cells—led to a hyperproliferation when compared to the control (124.6 ± 9.93% viable cells), Sunitinib treatment together with control transfections resulted in 71.33 ± 15.68% cell viability (Figure 3C). miR-221 overexpression in combination with Sunitinib administration did not significantly alter the viability in comparison to control transfections (75.13 ± 16.45%, *p* = 0.79). In line with these findings, a response toward Ki8751 was not significantly altered by miR-221 levels, either from 31.63 ± 11.7% in the control transfected to 32.53 ± 11.38% (*p* = 0.93, Figure 3D). 

Regarding the braking effect of miR-221 overexpression on VEGFR2 inhibition in PC3 cells (Figure 3A,B), we also employed Caspase 3/7 assays (Figure 3E). Exclusive miR-221 overexpression and exclusive Sunitinib administration (10 µM, 12 h) revealed the highest Caspase 3/7 activities compared to the control (240 ± 36.1% and 186.7 ± 5.8%). In line with our previous viability assays, a combination of miR-221 overexpression and Sunitinib caused a significantly lower Caspase 3/7 activity when compared to single Sunitinib administration (160 ± 10% vs. 186.7 ± 5.8%, *p* = 0.025).

We were further interested in Sunitinib-induced gene signatures in PCa cells. By searching L1000 Fireworks Display, which is a freely accessible database on gene signatures induced by small-molecule compounds, we obtained the gene signature of PC3 cells treated with Sunitinib (10 µM) for 6 h (ID: CPC014_PC3_6H:BRD-K70511574-001-01-0:10). We further examined this signature of *n* = 272 significantly upregulated genes and Reactome 2016 analysis (Figure 3F) revealed a strong upregulation of immune-related signaling pathways. As our group previously has shown, miR-221 overexpression in PC3 provoked a similar signature of interferon-related genes [12]. We looked for a Sunitinib-caused modulation of miR-221 and miR-222 expression in PCa cells. Strikingly, qRT-PCR experiments (Figure 3G) revealed a highly significant upregulation of miR-221 in PC3 cells treated with Sunitinib (10 µM) for 6h from 4.14 ΔC(t) in untreated PC3 cells to 6.88 ΔC(t) after Sunitinib administration (*p* = 0.0013) with 2.74 ΔΔC(t) representing a more than six-fold upregulation of miR-221 expression after Sunitinib treatment. For miR-222, Sunitinib treatment induced a significant upregulation of 1.18 ΔΔC(t) (*p* = 0.019, Figure 3G).

## 4. Discussion

Proangiogenic and specifically VEGFR2-mediated signaling crucially contributes to the high-risk setting in PCa [1,2]. For instance, Huss et al. showed that higher expression levels of VEGFR2 in PCa were associated with progressive disease [3]. In contrast, clinical trials focusing on VEGFR2 inhibition failed to show convincing results in PCa patients [27]. New biomarkers could help solve this dilemma by personalizing cancer therapy and identifying PCa patient subgroups likely to benefit. With this in view, miRs could be suitable candidates, as several studies have shown their functional relevance and their stage-specific expression in cancer. Moreover, the role of miRs within angiogenesis regulation is already well established [28,29]. For miR-221, regulation of KIT, a tyrosine kinase, and a target structure of Sunitinib has been demonstrated in endothelium biology and pathophysiology [28]. Additionally, members of our research group could show that KIT is a specific miR-221 target gene in PCa cells [8]. 

### 4.1. miR-221 as a Biomarker Candidate in TKI Therapy

In ccRCC patients, expression levels of miR-221 and miR-222 significantly predicted the response to TKI therapy in two different studies [6,7]. On a functional basis, this effect was explained by miR-221 and miR-222 targeting VEGFR2 [7]. By applying Luciferase reporter assays and Western blotting experiments, we could confirm this functional role of miR-221 in PCa cells. However, as indicated by miR-221 and VEGFR2 expression data from the TCGA cohort, VEGFR2 clearly does not have a stable and unidirectional role during oncogenesis and PCa progression, as its expression on average appears downregulated in PCa tissue. In line with this assumption, positive Pearson correlation coefficients for VEGFR2 and the established miR-221 target genes SOCS3 and IRF2, although statistically significant, appeared relatively low in our TCGA analysis. 

In contrast, examining our in-house high-risk PCa cohort and the multi-institutional Dream Team cohort consisting of PCa metastases [25] clearly showed a relevant VEGFR2 upregulation within the clinical high-risk setting, which confirms previous reports claiming a functional role of VEGFR2 in aggressive PCa [3,30]. Moreover, Nordby et al. claimed a prognostic influence of VEGFR2 expression on biochemical and clinical progression in a Norwegian PCa cohort [4].

A further potential mechanism of miR-221-mediated angiogenesis restriction is the strengthening of interferon signaling by targeting the functional repressors IRF2 and SOCS3 [12]. In general, interferon-mediated signaling is a known angiogenesis-limiting factor [31,32]. Furthermore, TNFSF10, which encodes for the Tumor Necrosis Factor Related Apoptosis Inducing Ligand (TRAIL), has been shown to counteract angiogenesis induced by VEGF [33]. In line with these findings, we could recently demonstrate that restoration of miR-221 strengthened TRAIL-mediated apoptosis in PCa cells [17].

In conclusion, regarding the results of this manuscript, our previous results and the established role of miR-221 in TKI resistance [7,34] together could offer a promising clinical perspective. Low miR-221 expression in aggressive PCa characterized by VEGFR2 overexpression not only marks patients with a significantly higher risk of progression [12], but those most likely to benefit from anti-angiogenic therapy.

### 4.2. miR-221 Upregulation as Part of a Sunitinib Escape Mechanism in PCa Cells

Given the VEGFR2 targeting of miR-221 in PCa cells, we next investigated a possible miR-221-mediated modulation of TKI sensitivity. Remarkably, proliferation assays confirmed a significant de-sensitization toward VEGFR2 inhibition in PC3 cells overexpressing miR-221. This effect was significant for both compounds Sunitinib and the VEGFR2 inhibitor Ki8751. This partially oncogenic function of miR-221 after VEGFR2 inhibition was also supported by Caspase 3/7 assays yielding a significantly lower apoptosis induction in miR-221 overexpressing PC3 cells treated with Sunitinib.

At first sight, the oncogenic role of miR-221 in Sunitinib escape appears controversial as we could show tumor suppressive effects of miR-221 in PC3 and DU145 cells before [12,17]. However, it has been shown that miRs exert stage-specific and microenvironment-dependent roles [35]. Regarding specific oncogenic functions of miR-221 and miR-222, both miRs were shown to be upregulated in castration-resistant subclones of LNCaP cells (LNCaP-Abl) [36]. Functionally, miR-221 targeted HECTD2 and RAB1A in castration-resistant LNCaP subclones, which modulates androgen receptor-mediated signaling and favors the progression to a castration-resistant state [14]. With this in view, miR-221/miR-222 overexpression as part of an escape from VEGFR2 inhibition could be well in line with publications demonstrating oncogenic roles of miR-221 and miR-222 in PCa cells [13,14,15,16]. 

We did not find similar miR-221 effects in LNCaP cells. When trying to address this discrepancy between PC3 and LNCaP, three major differences of these cell lines seem to stand out. First, LNCaP cells are castration-sensitive and have a lower metastatic potential, which, thereby, reflects earlier stages of PCa progression. Given the crucial role of miR-221 in androgen independence of PCa cells [14,36], it surely merits further investigation whether castration dependence determines the role of miR-221 in order to escape from TKI treatment. 

Second, LNCaP cells are unresponsive toward the interferon due to a lack of Janus kinase 1 (JAK1) [37], which is a trait especially relevant given that Sunitinib induced an interferon-related signature in PC3 cells. 

Moreover, the cellular PTEN status might serve as a third relevant difference between LNCaP and PC3 cells as the PI3K/Akt inhibiting gene PTEN was shown to be a direct target of miR-221 [38]. In contrast to PTEN-null PC3 cells, LNCaP cells show a PTEN mutation [39]. However, PTEN loss is significantly associated with higher Gleason scores and PCa progression [40]. In line with these findings, PI3K/Akt signaling is not only a major player in PCa progression but is also downstream of VEGFR2 signaling. Remarkably, we recently could demonstrate that miR-221 overexpression counteracted PI3K/Akt signaling in PC3 cells by targeting PIK3R1 [17].

### 4.3. Potential Implications for the Sequence of Anti-Angiogenesis and Immune-Based Approaches

For Sunitinib, we could demonstrate a new pro-immunogenic trait by inducing miR-221 expression in PCa cells. It is tempting to assume that the interferon-related gene signature in PCa cells after Sunitinib administration is partially caused or supported by miR-221 upregulation, which would be completely in line with an miR-221-mediated strengthening of JAK/STAT signaling in PCa shown by our research group [12]. Although several reports previously claimed a pro-immunogenic role of Sunitinib, this effect was mainly based on influencing immune cells such as regulatory T cells [41] and myeloid suppressor cells [42]. 

In terms of an ideal sequence therapy when combining TKI and immune-based approaches [43], our data potentially support the primary use of TKI with subsequent higher immune responsiveness of tumors due to higher miR-221 expression levels. These are first preliminary data and more research is needed to clarify the role of Sunitinib and miR-221/miR-222 in PCa as well as in other entities like RCC. 

## 5. Conclusions

Our results suggest a potential theragnostic window, which highlights that high-risk PCa patients with a significant downregulation of miR-221 in tumor tissue could be suitable candidates for an antiangiogenic therapy due to a VEGFR2 upregulation. We identified an oncogenic function of miR-221 as part of an escape mechanism after VEGFR2 inhibition in vitro. Although further research is needed, these results could be clinically relevant for sequential therapies by combining angiogenesis inhibitors and immune-based approaches.

## Figures and Tables

**Table 1:** Characteristics of our high-risk prostate cancer (PCa) cohort.

*n*	142
age at surgery	66 (47–81)
initial PSA (µg/L)	35.71 (20–597)
follow-up (months)	82.5 (1–154)
Gleason Score	
6	*n* = 3
7	*n* = 45
8	*n* = 46
9	*n* = 36
10	*n* = 12
pT stage	
2a	*n* = 4
2b	*n* = 14
2c	*n* = 3
3a	*n* = 41
3b	*n* = 59
4	*n* = 21
PSA progress	
yes (*n*)	*n* = 42
no (*n*)	*n* = 100
Clinical progress	
yes (*n*)	*n* = 20
no (*n*)	*n* = 122

“Age at surgery,” “initial prostate-specific antigen (PSA),” and “follow-up” variables are characterized as median values and an absolute range. pT stage: pathologic T stage.

**Table 2:** Characteristics of the prostate cancer (PCa) cohort within the TCGA (The Cancer Genome Atlas) database.

*n*	500
age at surgery	61 (41–78)
Gleason Score	
6	*n* = 45
7	*n* = 250
8	*n* = 64
9	*n* = 137
10	*n* = 4
pT stage	
2a	*n* = 13
2b	*n* = 10
2c	*n* = 165
3a	*n* = 159
3b	*n* = 136
4	*n* = 10
PSA progress	
yes (*n*)	*n* = 58
no (*n*)	*n* = 373
not assessed (*n*)	*n* = 69

“Age at surgery” is displayed as median value and absolute range. PSA: prostate-specific antigen; pT stage: pathologic T stage.

**Table 3:** Tumor entities with significantly aberrant VEGFR2/KDR expression in malignant tissue—according to the TCGA (The Cancer Genome Atlas) database, accessed via the UALCAN web resource.

Upregulated		Survival Impact	Downregulated	Survival Impact
Glioblastoma multiforme		ns	Cervical squamous cell carcinoma	ns
RCC, clear cell		**	Endometrial carcinoma	ns
Stomach adenocarcinoma		ns	Lung adenocarcinoma	ns
			Lung squamous cell carcinoma	ns
			PCa	ns
			RCC, chromophobe	ns
			RCC, papillary	**
			Urothelial carcinoma	ns

A significant impact on patients’ survival occurred in clear cell renal cell carcinoma (RCC) (upregulated) and papillary RCC (downregulated). PCa: prostate cancer. ns: not significant. **: *p* < 0.01.
